# Dry suction versus wet suction technique of endoscopic ultrasound-guided fine-needle biopsy for diagnosis of solid pancreatic lesions: study protocol of a multicenter randomized controlled non-inferiority trial

**DOI:** 10.1186/s13063-023-07842-8

**Published:** 2023-12-13

**Authors:** Shenglin Xu, Junfen Wang, Jianian Guo, Fang Xie, Weiguang Qiao, Yiteng Meng, Peng Peng, Haiyan Hu, Ying Huang, Side Liu, Mengbin Qin, Jun Yao, Yue Li

**Affiliations:** 1 Department of Gastroenterology, Nanfang Hospital, Southern Medical University, Guangzhou, China; 2 Department of Gastroenterology, Second Affiliated Hospital, Guangzhou Medical University, Guangzhou, China; 3 Department of Gastroenterology, Shenzhen People’s Hospital, Second Clinical Medical Sciences of Jinan University, Shenzhen, China; 4 Department of Gastroenterology, The Second Affiliated Hospital of Guangxi Medical University, Nanning, Guangxi China

**Keywords:** EUS-FNB, Franseen needle, Wet suction, Dry suction, Solid pancreatic lesions, Diagnostic yield

## Abstract

**Background:**

Studies have shown that the wet suction technique in endoscopic ultrasound-guided fine-needle aspiration (EUS-FNA) generates better histological diagnostic accuracy and specimen quality than the dry suction technique. However, conclusions of wet suction on the diagnostic accuracy of endoscopic ultrasound-guided fine-needle biopsy (EUS-FNB) are still controversial. Besides, the optimal number of passes for EUS-FNB has not been determined. We aimed to design a large multicenter randomized trial to compare the diagnostic accuracy of dry suction versus wet suction technique in solid pancreatic lesions (SPLs) using 22G Franseen needles and determine the optimal number of passes required for EUS-FNB.

**Methods:**

This is a multi-center open-label, randomized controlled non-inferiority trial with two parallel groups. Two hundred patients with SPLs will undergo EUS-FNB using 22G Franseen needles in 4 tertiary hospitals in China and will be randomly assigned to the dry suction group and wet suction group in a ratio of 1:1. The primary endpoint is diagnostic accuracy. Secondary endpoints include the optimal number of needle passes, sensitivity, specificity, specimen quality, cytological diagnoses, time of the procedure, and incidence of complications.

**Discussion:**

This study has been designed to determine (i) whether EUS-FNB using 22G Franseen needle with dry suction is non-inferior to wet suction in terms of diagnostic accuracy and (ii) the optimal number of passes during EUS-FNB of SPLs using 22G Franseen needle.

**Trial registration:**

ClinicalTrials.gov NCT05549856. Registered on September 22, 2022.

**Supplementary Information:**

The online version contains supplementary material available at 10.1186/s13063-023-07842-8.

## Background

Endoscopic ultrasound-guided fine-needle aspiration/biopsy (EUS-FNA/B) is an efficient diagnostic technique for solid pancreatic lesions (SPLs). Compared with FNA needles, the FNB needles can obtain much more core tissue, making histological investigations and genetic testing widely used, contributing to precision medication.

A novel wet suction technique for EUS-FNA was developed to improve the quality of aspirates and reduce blood contamination for diagnosis performance [1]. For the “wet suction” technique, the needle was flushed with saline solution to replace the column of air and then aspiration with a-10 ml pre-vacuum syringe. This is based on the principle that water is a less compressible fluid compared to air. Therefore, the volume of the vacuum could be enhanced when the EUS needle is filled with water (wet technique) [2]. A recent, randomized trial by Yun W. et al. has demonstrated that EUS-FNA using the wet technique is superior to the standard method in terms of diagnostic accuracy, sample adequacy, and sample blood contamination [3]. However, there are few studies on the impact of wet aspiration on the diagnostic accuracy of EUS-FNB. The sample size of these studies is small, and the conclusions are controversial.

The 2017 European Society for Gastrointestinal Endoscopy (ESGE) guidelines suggest the performance of two to three passes with an FNB needle when on-site cytologic evaluation is unavailable [4]. An RCT study demonstrated that the diagnostic accuracy of EUS-FNB alone with 3 needle passes in patients with SPLs reached 97.4% [5]. Of note, a recent large-scale multicenter RCT study showed that the overall diagnostic accuracy of FNB for SPLs was 94.7%, with an average of 2.81 passes required [6]. Unlike the reverse bevel needle, a needle with Franseen geometry was used in this study, and a meta-analysis showed that the Franseen needle was significantly superior to the reverse-bevel needles in both diagnostic accuracy and sample adequacy [7]. Further research is needed to explore the optimal needle passes of EUS-FNB using the Franseen needle.

The purpose of this multicenter RCT study is to compare the diagnostic accuracy of the dry suction technique versus the wet suction technique in SPLs using a 22G Franseen needle and determine the optimal passes required for EUS-FNB. This study may provide high-level evidence of EUS-FNB procedures for clinical practice.

## Methods

### Study design

**Fig. 1:**
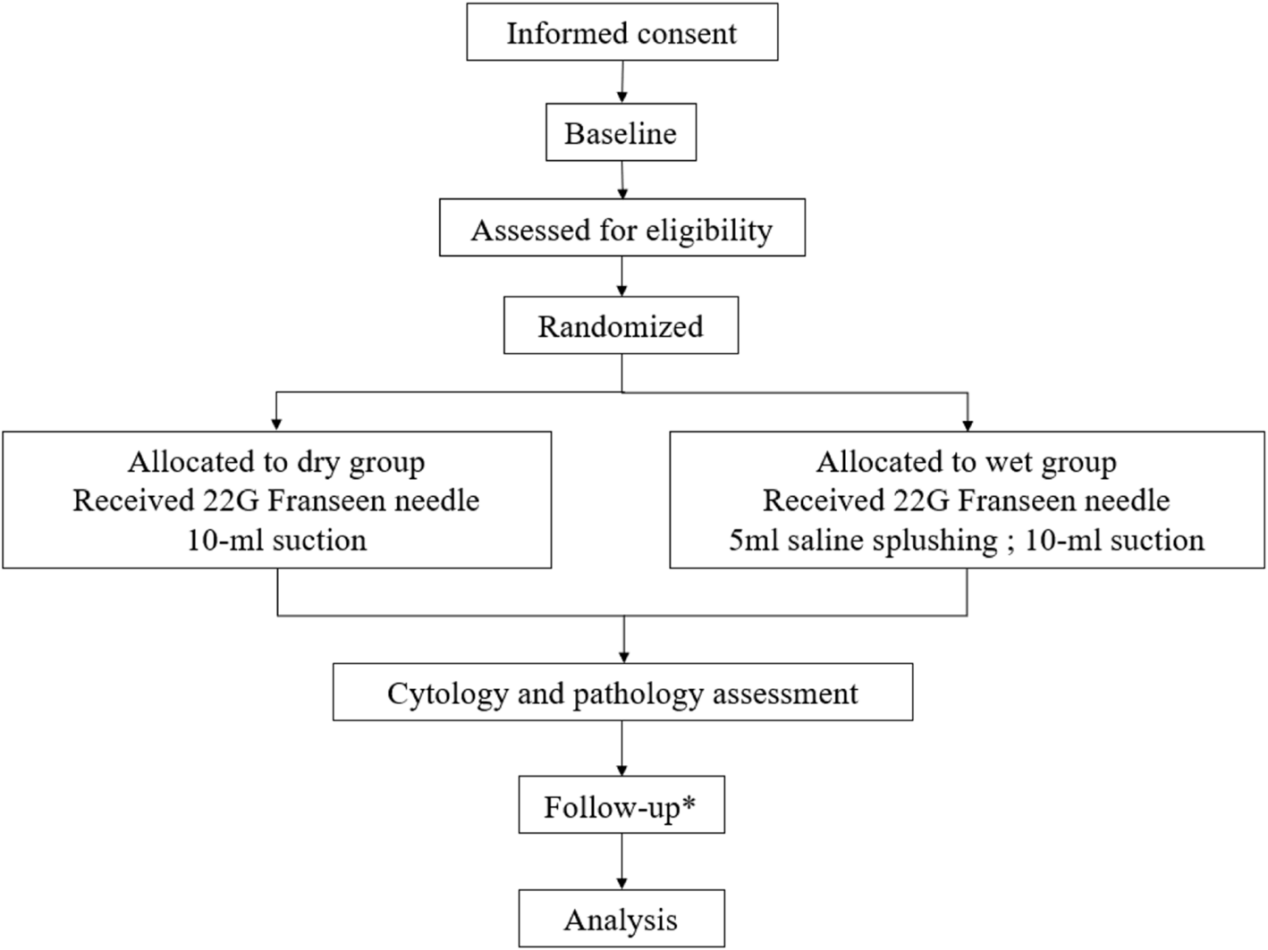
CONSORT flowchart. Asterisk (*) symbol indicates the following: we will follow-up with all patients for postoperative complications after the procedure within 24 h and 72 h. If the pathologic diagnosis of EUS-FNB samples is obtained during the punctures, we will observe at follow-up 1 year later

**Fig. 2:**
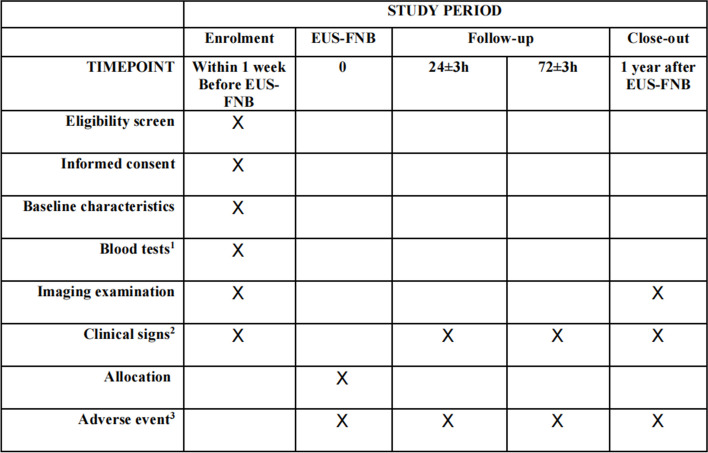
Standard protocol items Recommendations for Interventional Trials (SPIRIT): schedule for data collection. Superscript digit one (^1^) indicates the following: blood tests to be performed include blood routine test, total bilirubin, direct bilirubin, indirect bilirubin, INR, and CA-199. Superscript digit two (^2^) indicates the following: clinical signs include pain, weight loss, jaundice, etc. Superscript digit three (^3^) indicates the following: adverse events include unfavorable events and adverse reactions not related to the EUS-FNB procedure

This is a multicenter, open-label, randomized controlled non-inferiority trial with two parallel groups. Two hundred patients with SPLs will undergo EUS-FNB using 22G Franseen needles in 4 tertiary Chinese hospitals and will be randomly assigned to the dry suction group and the wet suction group in a ratio of 1:1. A checklist with the recommendations for Interventional Trials (SPIRIT) is attached as Additional file 1. Study flowchart and standard protocol items displaying the trial’s enrollment, intervention, and assessment schedules were showed in Figs. 1 and 2, respectively.

### Ethics approval and patient consent

The study was approved by the ethics committee of NanFang Hospital of Southern Medical University (NFEC-2022–335) on 1 September 2022. Subsequently, the ethics committee of each participating hospital approved the trial. At each participating center, the study investigators will obtain informed consent from the candidates. The trial was registered at Clinical Trials.gov with identifier No. NCT05549856.

Participants will provide informed consent to the sampling of biological material(pancreatic lesions), which will be deidentified and stored in the secure research facilities of the participating trial sites. Consent for the necessary processing of the samples such as medical photography and preservation, pathology examination, and medical waste disposal is addressed.

### Outcomes

The primary outcome is to compare the rate of diagnostic accuracy of EUS-FNB using the two different suction techniques (dry suction and wet suction). The rate will be calculated as the sum of true positive values and true negative values divided by the total number of samples, with an accurate diagnosis defined as a final diagnosis consistent with the tissue sample obtained during the needle passes.

The secondary outcomes include the optimal number of needle passes, sensitivity, specificity, specimen quality, cytological diagnoses, time of the procedure, and incidence of complications. Specificity analysis is only applicable to participants without malignancy, whereas sensitivity analysis is only applicable to persons with malignancy.

### Eligibility criteria

#### Inclusion criteria

The inclusion criteria for trial eligibility are as follows:

- Age from 18 to 85 years
- Diagnosis or suspicion of a solid pancreatic lesion based on previous imaging examination (ultrasonography, computed tomography [CT], or magnetic resonance imaging [MRI])
- Written informed consent obtained

#### Exclusion criteria

The exclusion criteria are the following:

- Any condition that could prevent endoscope insertion or EUS-FNB procedure
- That no pancreatic lesion on EUS
- Use of anticoagulants/antiplatelet drugs that cannot be discontinued
- Coagulation disorder: international normalized ratio (INR) > 1.5 or platelet counts < 50,000/mm^3^
- Pregnancy or patients with mental disorders
- Other medical conditions make the patient unsuitable for ineligibility
- Inability or refusal to provide informed consent

### Randomization and blinding

Patients will be evaluated by gastroenterologists, surgeons, oncologists, and endoscopists. The potential candidates will be informed and enrolled in this trial by our study team. Stratified blocked randomization was used in the present study. The list of random numbers was generated by an independent statistician who was blinded to this study using the R software (v 4.0.3) by block with stratification on gender. The patients will be allocated randomly to either the dry suction group or the wet suction group in a ratio of 1:1 according to random numbers. To prevent different subject recruitment rates at the various hospitals from interfering in the development of the study, each center will use its list, and the entire population will be randomized in blocks between the two groups.

We used sequentially numbered sealed opaque envelopes to conceal allocation. The cytologists and the pathologists will be blinded during the entire study.

### Participating sites

All four sites are large tertiary hospitals in China. To avoid biases derived from the learning curve, only the sites with annual EUS-FNA/FNB cases ≧ 100 have been invited to participate in the present study. Endoscopists experienced in interventional EUS, such as EUS-FNA/FNB more than 100 cases, performed the procedures. The Nanfang Hospital of the Southern Medical University is key in minimizing heterogeneity and centralizing decision-making in conflicts. During the conduct of the trial, the principal investigators will monitor the enrollment targets monthly, and the coordination meeting was organized periodically.

### EUS-FNB procedure

Patients will be assigned to group A (dry suction) or group B (wet suction). In both groups, we will use the 22G Franseen needle (Acquire™, Boston Scientific) to puncture the SPLs.

All procedures will be conducted by doctors with experience in more than 100 cases of EUS-FNA/FNB before the start of the study. During the procedure, the needle was moved back and forth about 10–20 times using the fanning method as much as feasible. Standard negative pressure with a 10-mL syringe was applied in both groups.

### Dry suction

In the dry group, before puncturing the lesion, the stylet is removed, and a 10-ml air-filled pre-vacuum syringe is attached. The needle is moved back and forth about 10–20 times within the lesion using a fanning technique. Afterward, the needle is withdrawn from the lesion.

### Wet suction

With the use of the wet suction technique, the stylet will be removed from the needle before puncture; the needle will then be flushed with 5 mL of saline to replace the column of air with fluid. After the needle punctures the lesion, a 10-mL air-filled syringe will be attached to the end of the needle to provide continuous negative pressure suction while the needle is moved back and forth.

### Post-trial care

In the postoperative period, we will make a 72-h follow-up to monitor any postoperative adverse events. The participants will be compensated with $100 when they finish the trial and follow-up.

### Specimen

After 3 needle passes, the doctor will evaluate the adequacy of the sample with macroscopic on-site evaluation (MOSE) and decide whether to continue additional punctures. MOSE was performed by gross visualization of the collected material by the endoscopists and considered adequate when a white/yellowish aggregate core longer than 10 mm was retrieved. Three needle passes at least were required for both groups. The specimens obtained at each pass will be marked and sent to pathology separately. It is to be evaluated using traditional cytological smear, liquid-based thin layer cytological examination, and histological examination for each pass.

### Cytological and histological analyses

Histopathological diagnosis will be based on cytology or histology. Specimens fixed with formalin will be embedded in paraffin blocks. Immunostaining and nuclear staining will be performed as needed. Experienced pathologists who have performed at least 1000 cytology and histology evaluations of EUS-FNA/B specimens will assess the specimens independently. The pathologists will be blinded to the allocation.

### Final diagnosis

The malignant diagnosis was defined as the cytological or pathological diagnosis considered suspicious or positive for malignancy. Neuroendocrine tumors, solid pseudopapillary neoplasm, lymphoma, and melanoma were also considered to be malignant lesions.

The final diagnostic standards are as follows:

1. For patients who undergo surgery, the final diagnosis will be based on the definite evidence of malignancy from surgical specimens.
2. For patients who did not receive surgery, the final diagnosis will be considered as malignancy if cytologic or histologic diagnosis of malignancy is made by EUS-FNB with a corresponding clinical manifestation.
3. For patients with negative or unsatisfactory pathological results, the final diagnosis will be confirmed by clinical and imaging follow-up for 1 year. If there was no imaging progression of the SPL, the true negative would be considered, and if there was progression or death, the false negative would be determined.

### Data collection and management

The data will be collected using case record forms (CRF) by trained doctors. Figure 2 shows the schedule of enrollment, interventions, and assessments. We will conduct regular quality checks and validation procedures and implement strict protocols for data security and storage to ensure data quality, security, and storage.

Our study team will make maximum effort to have participants complete the study follow-up assessments. The study team will contact participants via telephone to collect as much outcome data as possible. All data obtained until the time of withdrawal will be retained in the study.

A comprehensive data management plan is implemented to ensure data quality, security, and storage. This includes using standardized CRF templates and guidelines for data entry, conducting regular quality checks and validation procedures, and implementing strict protocols for data security and storage.

Biological specimens will be collected during the surgical procedures and used for routine diagnostic tests. The biopsy samples will be stored in the Pathology Department of the hospital. No genomic or molecular analysis are planned in the current trial or for future use.

### Confidentiality

Only the informed consent forms will contain patient names and will be stored locally in a safe location at each participating center. Personal data about participants is retained using an identifier in the CRF. Only the authorized research team will be granted access to personal information about participants.

### Sample size calculation

The sample size calculation was based on the primary outcome of diagnostic accuracy for the evaluation of solid pancreatic lesions. The reported diagnostic accuracy of EUS-FNB for SPL using dry suction is approximately 96.2%, and the wet suction technique is about 98.3% in the literature [6, 8]. Based on the reported data and the clinical judgment, the non-inferiority of dry suction in EUS-FNB will be assessed with a non-inferiority (*δ*) margin of 10%. With a two-sided test, a significance level of 2.5% (*α* = 0.025), and a power (1 − *β*) of 90%, using the PASS 15.0 software and a sample allocation ratio of 1:1 between the dry suction and wet suction groups, 180 patients should be enrolled. Considering a 10% dropout rate, a final sample of 200 participants (100 per group) is needed.

### Statistical analysis plan

The full analysis set should be as close as possible to the intention-to-treat set. The direct deletion method will be used to treat missing data.

Continuous variables will be summarized as means with standard deviation and compared with *t*-tests or Wilcoxon rank-sum tests. Categorical variables will be expressed as percentages using the *χ*^2^ test or the Fisher exact test. Differences in diagnostic yield between the two techniques will be evaluated with the chi-squared test. Statistical significance will be considered for *P* < 0.05. All statistical analyses will be performed using SPSS (version 25.0, IBM, Armonk, NY).

### Monitoring

A data monitoring plan and detailed routine verifications of the CRFs will be proposed. An independent data monitoring committee (DMC) is established to ensure the safety and efficacy of the trial. The DMC will regularly review the progress of the trial, including recruitment, data collection and analysis, serious adverse events, etc., and provide advice to improve the quality.

If an adverse event occurs during the trial, the investigator must record the adverse event form, including the time, severity, duration, measures taken, and regression of the adverse event, and report to the ethics committee and regulatory authorities in time.

If an important modification to the study protocol is required for any reason, it will be approved in advance by the ethical committee. Investigators and participants will be notified of any significant protocol revisions.

### Publication of results

The trial results will be presented as abstracts at international gastrointestinal meetings, such as Digestive Disease Week and United European Gastrointestinal Week. We will publish the final data in a peer-reviewed medical journal to expand the dissemination.

## Discussion

There are several variables affecting the outcomes of EUS-FNA/FNB, including needle selection, operator experience, adequacy of sample, use of suction, the number of needle passes, and the presence of ROSE. Increasingly studies have reported that the Franseen needle can provide higher diagnostic yield and accuracy compared to other needle types, particularly for SPL [7, 9, 10]. The wet suction technique in EUS-FNA has been proven to reduce blood contamination and increase diagnostic yield [2, 3]. An RCT study found that EUS-FNB with wet suction resulted in a significantly better quality of specimen, histological, and first-pass diagnostic yields compared with the dry suction, but they used 5 ml negative pressure, which differed from the standard negative pressure previously reported [11]. Another study has shown that EUS-FNB using the slow pull, wet technique, or standard technique makes no significant difference in terms of specimen quality or the number of needles required to obtain a diagnosis. However, the study was a single-center study which was early terminated, resulting in inadequate sample size and convincing data [12].

In the previous RCT, dry suction versus wet suction was only compared during EUS-FNA procedure. There was no data for EUS-FNB using Franseen needle. In our study, we will compare the diagnostic accuracy of dry suction versus wet suction technique, and we also explore the optimal numbers of passes for SPLs. Smaller numbers of needle passes will be beneficial to patients in terms of procedure time, unnecessary passes, and risk of complications. We chose a non-inferiority design based on the expectation that dry suction of EUS-FNB is as good as wet suction and the dry suction can simplify the procedure and shorten the procedure time. With the improved performance of needles, satisfactory diagnostic accuracy of EUS-FNB in SLPs may be achieved only by dry suction and limited needle passes.

This study design has some limitations. First, this study will be performed in a multicenter setting; the bias between operators may still exist despite the training on standardized processes for all centers. Large sample size and randomization have been used to counteract this possible bias and to obtain results that allow for extrapolation to the general population.

Second, surgical pathological data was not available in all patients with SPLs. Some patients do not undergo surgery because the lesion is benign or because they refuse surgical treatment. Thus, clinical and imaging follow-up will be done for at least 12 months to determine the final diagnosis.

In conclusion, this is the first multicenter randomized controlled trial designed to clarify whether the dry suction technique is non-inferior to the wet suction technique and the optimal needle passes for SPLs using a 22G Franseen needle. The findings may provide high-level evidence of EUS-FNB procedures in clinical practice.

## Trial status

Protocol version number: 1.0, May 26, 2022.

Patient recruitment was initiated in September 2022 and is estimated to be completed by June 2024. At present (October 13, 2023), 123 patients have been enrolled in the study.

### Supplementary Information

**Additional file 1.**

## Data Availability

The case report form is stored in the Nanfang Hospital. The individual data of each patient will be stored in each center and will not be available to the public because of “Protection of Personal Data.” The principal investigators will be given access to the cleaned datasets, and the data can be available via contacting PI if requested.

## Abbreviations

EUS: Endoscopic ultrasonography
EUS-FNA: Endoscopic ultrasound-guided fine-needle aspiration
EUS-FNB: Endoscopic ultrasound-guided fine-needle biopsy
FNA: Fine-needle aspiration
SPLs: Solid pancreatic lesions
CT: Computed tomography
MRI: Magnetic resonance imaging
INR: International normalized ratio
CA-199: Carbohydrate antigen 19–9
MOSE: Macroscopic onsite evaluation
